# Robotic spine surgery: seeing is believing

**DOI:** 10.3171/2025.4.FOCVID24146

**Published:** 2025-07-01

**Authors:** Nicholas Theodore, Ali Baaj, Brandon Carlson, Nicolai El Hindy, Karthik Madhavan, Anand Veeravagu, Laura Snyder

**Affiliations:** 1 Department of Neurosurgery, Johns Hopkins School of Medicine, Baltimore, Maryland;; 2 Department of Neurosurgery, Banner-University Medical Center Phoenix, Arizona;; 3 Department of Orthopedic Surgery and Sports Medicine, University of Kansas Medical Center, Kansas City, Kansas;; 4 Department of Neurosurgery, St. Marien Hospital Lünen, Germany;; 5 Department of Neurosurgery, Cooper University Healthcare, Camden, New Jersey;; 6 Department of Neurosurgery, Stanford University School of Medicine, Stanford, California; and; 7 Department of Neurosurgery, Barrow Neurological Institute, Phoenix, Arizona

When Czech writer Karel Capek introduced the word "robot" in his 1920 science fiction play *R.U.R.*,^1^ the concept conjured up a frightening possibility of rogue devices that could end the human race. Thankfully, the development of robots has been confined to assisting humans in tasks from manufacturing to butchery. Always slow to adopt technology, the field of surgery has been the beneficiary of these exciting technologies. While navigation in spinal surgery is not a new concept and was initially introduced to improve accuracy with pedicle screw placement, the addition of robotics has taken our field to the next level. Instead of describing these cutting-edge techniques in dry prose, this issue uses powerful video demonstrations of a panoply of different robots and their usage in spinal surgery. Seeing is believing, and this compendium represents a meaningful example of the speed at which technology is adopted and modified for various uses in our field. No longer entities to be feared, robots are here to stay. Understanding their usage and value in spinal surgery is critical as they become part of our armamentarium in the future.

## Disclosures

Dr. Theodore reported personal fees from Globus Medical; in addition, he had a patent for 11,103,317 B2 licensed. Dr. Baaj reported consulting for J&J. Dr. Carlson reported personal fees from Globus Medical and Amgen, and stock in SpinEM Robotics. Dr. El Hindy reported being a robotic user. Dr. Madhavan reported being a teaching consultant for Stryker/VB Spine. Dr. Veeravagu reported being a consultant with royalties for ATEC, Globus, and OsteoCentric. Dr. Snyder reported consulting fees from Globus.
